# Microbiological Relevance of *Candida* in Urine Cultures

**DOI:** 10.3390/jof11070483

**Published:** 2025-06-26

**Authors:** Leticia Castellano-Sánchez, Antonio Rosales-Castillo, Raquel Marcos-Rodríguez, María Carmen Olvera-Porcel, José María Navarro-Marí, José Gutiérrez-Fernández

**Affiliations:** 1 Microbiology Laboratory, Hospital Universitario Virgen de las Nieves, Instituto de Investigación Biosanitaria de Granada, 18012 Granada, Spain; leticia.castellano.sspa@juntadeandalucia.es (L.C.-S.); raquel.marcos.rodriguez.sspa@juntadeandalucia.es (R.M.-R.); josem.navarro.sspa@juntadeandalucia.es (J.M.N.-M.); 2 Clinical Medicine and Public Health Doctoral Program, PostGraduate School, University of Granada, 18012 Granada, Spain; 3 Internal Medicine Department, Hospital Universitario Virgen de las Nieves, Instituto de Investigación Biosanitaria de Granada, 18012 Granada, Spain; 4 Biostatistics, Unidad de Gestión y Apoyo a la Investigación, Hospital Universitario Virgen de las Nieves, 18014 Granada, Spain; mariac.olvera.sspa@juntadeandalucia.es; 5 Department of Microbiology, Faculty of Medicine, University of Granada, Instituto de Investigación Biosanitaria de Granada, 18016 Granada, Spain

**Keywords:** *Candida*, urine, hospitalization, intensive care, urinary catheter, urine culture

## Abstract

The presence of *Candida* spp. in urine has traditionally been considered to be a colonization; however, in certain clinical circumstances, such as in critically ill patients, immunocompromised individuals, or those with chronic diseases, it gains greater relevance due to the possibility of active infection and complications. The aim of this study was to characterize the epidemiology (incidence, species distribution, demographic characteristics, and origin) of *Candida* spp. isolates in urine through a retrospective cross-sectional analysis of urine culture isolates from clinical samples between January 2016 and December 2023. Out of a total of 111,656 urine cultures, *Candida* spp. was isolated at 2.72% (*n* = 3037). The most frequently isolated species was *Candida albicans* (54.25%; 1646/3037), followed by *Nakaseomyces glabrata* (22.78%; 692/3037) and *Candida tropicalis* (10.2%; 311/3037). Advanced age (>70 years), male sex, presence of a urinary catheter, and origin from intensive care units, oncology, or surgical services were variables associated with a higher risk of candiduria, highlighting the relevance of candiduria in the presence of such clinical scenarios.

## 1. Introduction

Urinary tract infections (UTIs) caused by *Candida* spp. have become an issue of growing concern in hospital settings. Traditionally, candiduria has largely been regarded as an asymptomatic colonization; however, recent evidence suggests that its detection is gaining relevance across various clinical contexts and conditions, such as chronic diseases, immunosuppressed states, or intensive care units, where it may be associated with active infections and significant clinical complications [1,2]. In this regard, studies have emphasized that beyond its mere presence, the pathogenic role of *Candida* spp. in the urinary tract should be assessed and individualized based on specific risk factors and the patient’s immune status.

Heras-Cañas et al. [3] described the diversity of yeast species isolated from urine samples, highlighting not only the predominance of *Candida albicans* but also a rising incidence of non-albicans species, which in some cases exhibit antifungal resistance patterns. These findings have been corroborated by subsequent studies, such as that of Jiménez-Guerra et al. [4], which analyzed the etiology, antifungal susceptibility, and risk factors in hospitalized patients, underscoring the importance of accurate diagnosis to differentiate colonization from active infection.

In critically ill patients, the incidence of candiduria is particularly high. Álvarez-Lerma et al. [2] reported a high prevalence in intensive care units, suggesting that the presence of *Candida* spp. in the urinary tract may serve as a marker of severity in such settings. Likewise, Horcajada et al. [5] demonstrated that imaging techniques can detect upper urinary tract involvement in patients with candiduria, raising awareness of the need for more comprehensive clinical evaluation in certain cases.

The role of medical devices—particularly urinary catheters—has been widely recognized as a significant risk factor for the development of candiduria. Studies by Padawer et al. [6], Freitas et al. [7], and Al-Haifi et al. [8] have shown that catheterization is strongly associated with an increased incidence of infection, due to the disruption of natural barriers and biofilm formation on these devices. Moreover, accurate species identification, facilitated by advanced techniques such as mass spectrometry and molecular methods [9,10], is crucial for determining the therapeutic approach, especially in light of emerging antifungal resistance in some species [11,12]. The clinical complexity of candiduria is further compounded in populations with comorbidities, such as diabetic patients. Esmailzadeh et al. [13] and Akinjogunla et al. [14] have shown that candiduria in this group is not only more frequent but is also often caused by non-albicans species with more pronounced resistance profiles, posing additional therapeutic challenges. On the other hand, the progression of candiduria to systemic complications, such as candidemia, has been the subject of predictive modeling studies aimed at identifying patients at highest risk of systemic infection originating from candiduria [15].

The clinical management of candiduria remains controversial, largely due to the difficulty in distinguishing between asymptomatic colonization and active infection. While international guidelines, such as those from the IDSA [16], have proposed diagnostic criteria and treatment recommendations, their application in clinical practice varies depending on the context and patient population [1,17]. In this regard, the recent global guideline for the diagnosis and management of candidiasis [18] offers an updated framework emphasizing the need for individualized patient assessment. It highlights risk factors such as urinary catheter use, advanced age, and comorbidities to accurately differentiate colonization from true infection. This approach supports the recommendation of antifungal therapy only in cases with clear evidence of infection, thereby avoiding unnecessary interventions and contributing to antifungal resistance prevention, as also noted in previous studies [4,6].

Additionally, recent studies from different regions, such as those by Ghasemi et al. [19] and Altınbaş and Bildirici [20], have provided updated information on prevalence, species distribution, and antifungal susceptibility trends in hospitalized patients, underscoring the need for continuous surveillance and strategies tailored to local conditions.

Given this scenario, there is a pressing need for studies that comprehensively characterize the epidemiology of candiduria in specific hospital contexts. The present study aims to describe the incidence, species distribution, demographic characteristics, and sources of urine cultures positive for *Candida* spp., with the goal of identifying scenarios with the highest likelihood of isolation and providing valuable information to inform clinical decision-making, ultimately improving patient outcomes.

## 2. Materials and Methods

A descriptive, cross-sectional, and retrospective study was conducted, including all urine culture samples processed at the Microbiology and Parasitology Laboratory of the Virgen de las Nieves University Hospital in Granada between January 2016 and December 2023. This hospital is a regional healthcare complex comprising three centers (General Specialty Hospital, Mother and Child Hospital, and the Neurotraumatology and Rehabilitation Hospital) providing tertiary care services in the province. Given the single-center design, the findings may reflect local epidemiological and clinical characteristics, which could limit the external validity of the results.

The study did not include explicit data on urinary symptoms. Only the following variables were collected through the laboratory information system (MODULAB^®^, Werfen Laboratories, Barcelona, Spain), which supports the electronic medical record system of the Andalusian Public Health Service: sample source, microorganism, patient sex, and age. These were later analyzed, first comparing isolates other than *Candida* spp. and subsequently analyzing only the *Candida* spp. isolates.

All cases were distinct episodes, occurring at least six weeks apart. The only exclusion criteria were duplicated or repeated microbiological tests for the same episode. Urine samples, collected under conditions minimizing contamination and upon suspicion of UTI, included midstream clean-catch, indwelling catheter, intermittent catheterization, pediatric urine bag, and nephrostomy catheter. All samples were processed following standard procedures. Urine cultures were detected and interpreted using chromogenic media CHROMID^®^, CPSO^®^, and CHROMID^®^ *Candida* (bioMérieux, Marcy-l’Étoile, France) after 24–48 h incubation at 37 °C. Species identification was performed using MALDI-TOF mass spectrometry (Number 00556029, Biotyper, Bruker Daltonics, Billerica, MA, USA).

### 2.1. Ethical Approval and Consent to Participate

The study protocol was conducted in accordance with the Declaration of Helsinki and the ethical considerations of epidemiological research. This was a non-interventional study, with no further investigation into routine procedures. The biological material was used only for the standard diagnosis of infections as ordered by attending physicians. No additional sampling or modification of the routine diagnostic protocol was performed. Data analyses were performed using a completely anonymous database, where subjects were replaced at different infectious episodes, occurring at least 6 weeks apart from the previous one, if any. Permission to access and use the data was granted by the Clinical Microbiology Management Unit of Virgen de las Nieves University Hospital (Granada, Spain). Ethics committee approval was considered unnecessary according to national guidelines (Law on Data Protection—Organic Law 15/1999 of December 13 on the protection of data of a personal nature, https://www.boe.es/eli/es/lo/1999/12/13/15, accessed on 15 April 2025).

### 2.2. Statistical Analysis

Descriptive statistical analysis was performed. Quantitative variables were expressed as median and interquartile range (IQR), and categorical variables were summarized using frequency tables and percentages. Categorical variables were compared using the chi-square (χ^2^) test. The association of each independent variable with *Candida* spp. presence was evaluated using crude Odds Ratios (ORc). To assess the potential influence of confounding variables, a multivariate logistic regression model was constructed to calculate adjusted Odds Ratios (ORa). Variables were excluded from the final model based on the likelihood ratio test. Confidence intervals (CIs) were set at 95% for both ORc and ORa. The Hosmer–Lemeshow test assessed model fit, and the ROC curve was used for model discrimination. A *p*-value < 0.05 was considered statistically significant. Statistical analyses were conducted using STATA version 16.1.

## 3. Results

Between January 2016 and December 2023, a total of 111,656 urine cultures were analyzed, with patient ages ranging from 0 to 106 years. The median age was 59 years (IQR: 43). Of these, 12.79% (*n* = 14,285) were from pediatric patients (≤15 years), and 87.21% (*n* = 97,371) from adults. Women accounted for 54.20% (*n* = 60,516) and men for 45.80% (*n* = 51,140). A total of 26.26% (*n* = 29,320) of patients had indwelling urinary catheters.

Sample origin by requesting department was as follows: 44.72% from Emergency (*n* = 49,928), 40.17% from Medical Services (*n* = 44,851), 5.60% from ICU (*n* = 6248), 3.83% from Surgical Services (*n* = 4274), 2.33% from Outpatient Clinics (*n* = 2597), 1.75% from Oncology (*n* = 1953), and 1.62% from Primary Care (*n* = 1805) (Table 1).

Of the total urine cultures, 34.44% (*n* = 38,458) were positive, including both bacterial and fungal isolates. Bacterial isolates accounted for 31.72% (*n* = 35,421), while fungal isolates corresponding to *Candida* spp. accounted for 2.72% (*n* = 3037). The distribution of *Candida* species was as follows: *Candida albicans* (54.25%; 1646/3037), *Nakaseomyces glabrata* (22.78%; 692/3037), *Candida tropicalis* (10.2%; 311/3037), *Candida parapsilosis* (3.32%; 101/3037), *Pichia kudriavzevii* (2.14%; 65/3037), and *Clavispora lusitaniae* (1.51%; 46/3037). Other yeasts (e.g., *Meyerozyma guilliermondii*, *Kluyveromyces marxianus* (before *Candida kefyr*), *Pichia inconspicua* and *Debaryomyces hansenii* (before *Candida famata*)) were rare (<0.01%). An additional 4.37% (*n* = 133) were unidentified yeasts.

Bivariate analysis revealed significant differences between patients with and without *Candida* spp. isolation. Patients with candiduria were significantly older (median: 74 years; IQR: 22) than those without (median: 59 years; IQR: 42). Among pediatric patients, the prevalence of candiduria was lower (0.36%; 51/14,285) compared to adults (3.07%; 2986/97,371), *p* < 0.001.

Of patients with candiduria, 50.84% (*n* = 1544) were women. However, men had a slightly higher risk (2.92%) than women (2.55%), *p* < 0.001. Catheterized patients had a significantly higher prevalence of candiduria (6.12%; *n* = 1793) versus non-catheterized (1.51%; *n* = 1244).

Among yeast-positive samples, distribution by service was 50.08% from Medical Services (*n* = 1521), 27.69% from Emergency (*n* = 841), 11.89% from ICU (*n* = 361), 6.09% from Surgery (*n* = 185), 3.42% from Oncology (*n* = 104), 0.69% from Outpatient Clinics (*n* = 21), and 0.13% from Primary Care (*n* = 4).

Bivariate logistic regression showed that male sex increased the risk of candiduria (OR: 1.15; 95% CI: 1.07–1.23; *p* < 0.001). Compared with Primary Care, the risk varied by department: ICU (OR: 27.61; 95% CI: 10.29–74.07; *p* < 0.001), Oncology (OR: 25.33; 95% CI: 9.31–68.89; *p* < 0.001), Surgery (OR: 20.37; 95% CI: 7.55–54.94; *p* < 0.001), Medical hospitalization (OR: 15.80; 95% CI: 5.92–42.23; *p* < 0.001), Emergency (OR: 7.71; 95% CI: 2.89–20.62; *p* < 0.001), and Outpatient Clinics (OR: 3.67; 95% CI: 1.26–10.71; *p* < 0.001). Non-pediatric patients had a significantly higher risk (OR: 8.83; 95% CI: 6.69–11.65; *p* < 0.001). Urinary catheterization increased candiduria risk by over fourfold (OR: 4.25; 95% CI: 3.94–4.57; *p* < 0.001) (Table 2).

**Figure 1:**
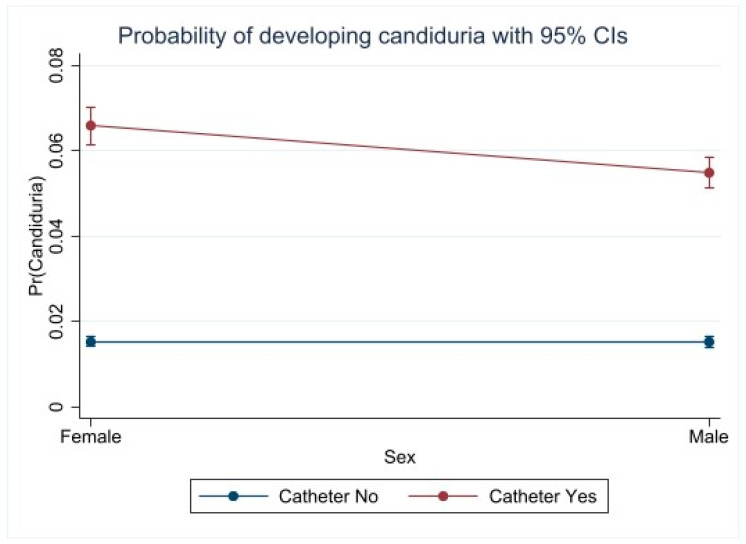
Probability of Candiduria in patients according to sex and urinary catheter use. It is observed that patients with a urinary catheter have a significantly higher probability of developing candiduria compared to those without a catheter. Within the group with a urinary catheter, women exhibit a slightly higher probability than men. In contrast, the probability of candiduria in patients without a urinary catheter remains low and invariant between sexes.

Regarding the variables that were ultimately included in the multivariable logistic regression model, a significant interaction was observed between sex and the presence of a catheter. Among patients without a catheter, there were no significant differences between men and women regarding the odds of having *Candida* spp. (adjusted OR: 1.00; 95% CI: 0.89–1.12; *p* = 0.99). However, among patients with a catheter, men had a lower probability of *Candida* spp. infection compared to women with a catheter (adjusted OR: 0.82; 95% CI: 0.74–0.90; *p* < 0.001). Additionally, catheter use was strongly associated with *Candida* spp. infection in both sexes. Compared to patients without a catheter, the odds were significantly higher in women (OR: 4.61; 95% CI: 4.15–5.12; *p* < 0.001) and men (OR: 3.78; 95% CI: 3.38–4.22; *p* < 0.001) (Figure 1).

Non-pediatric patients [1] were significantly more likely to experience the outcome compared to pediatric patients (OR: 11.83; 95% CI: 8.96–15.63; *p* < 0.001). Regarding the type of clinical service, primary care was used as the reference category. Compared to it, higher odds of the outcome were observed across all other services: oncology (OR: 15.06; 95% CI: 5.52–41.04), medical wards (OR: 9.49; 95% CI: 3.55–25.38), surgical wards (OR: 8.77; 95% CI: 3.25–23.71), ICU (OR: 10.05; 95% CI: 3.73–27.04), emergency department (OR: 5.04; 95% CI: 1.88–13.49), and outpatient clinics (OR: 3.07; 95% CI: 1.05–8.96).

The model demonstrated acceptable discrimination (AUC = 0.7457).

## 4. Discussion

This study analyzes the overall prevalence of candiduria in both hospitalized and outpatient populations. Outpatients accounted for only 0.82% of the *Candida* spp. isolates, indicating that its clinical significance is primarily confined to the hospital setting. In a previous study [21], it was estimated that up to 9.4% of healthcare-associated urinary tract infections (UTIs) were caused by *Candida* spp. Moreover, up to 18% of UTIs in patients with indwelling urinary catheters and 1% in critically ill patients have been attributed to *Candida* spp. [22]. Its incidence is likely underestimated due to several factors. From a diagnostic standpoint, microbiological variables may interfere with *Candida* spp. identification in urine, including the slower growth of non-albicans *Candida* species (more than 48 h) on chromogenic media [23], the limited sensitivity of standard urine cultures—especially for non-albicans *Candida*—and the reduced detection capabilities of flow cytometry techniques, particularly for small colony-forming species such as *N. glabrata* [4].

The highest prevalence was observed in Intensive Care Units (ICUs), ranging from 20 to 30% among patients with hospital stays longer than seven days, most of whom have indwelling urinary catheters. Colonization or infection typically occurs later during hospitalization; a French ICU study reported an average of 17.2 ± 1.1 days until onset [24]. Among kidney transplant recipients, this figure increased to 54 days post-transplant [25].

The described risk factors include the presence of urinary or nephrostomy catheters, urinary tract abnormalities, abdominal surgery, broad-spectrum antibiotic use, female sex, age over 65 years, diabetes mellitus, immunosuppression, mechanical ventilation, and ICU stay.

Findings from our study regarding age are consistent with the literature [2], showing a median age of 74 years in patients with candiduria compared to 59 years in those without. In the pediatric population, only 51 isolates (0.36% of the total) were identified—significantly lower than the 3.07% found in adults—supporting age as a risk factor. In children, most cases were associated with oncohaematological disorders, ICU stay, antibiotic therapy, and indwelling urinary catheters [10,26]. No sex-related differences were observed (49.16% male, 50.84% female).

Urinary catheter use was reported in 59.04% of *Candida* spp. isolates, which is notably higher than in non-*Candida* isolates (25.34%), aligning with some previous studies [27], though lower than in others [4]. *C. albicans* is recognized as the second most common etiological agent of catheter-associated UTIs after *Escherichia coli*. This is clinically relevant, as catheter removal alone can result in candiduria resolution in over 40% of patients, and catheter replacement in up to 20% [28].

Consistent with other reports [4,17,29], *C. albicans* was the most frequently isolated species (54.25%), within the commonly reported range of 50–70%. *N. glabrata* and *C. tropicalis* followed in frequency; these three species are the most frequently documented in the literature [30]. However, the rise in non-*albicans Candida* species in recent decades must be considered [31,32], as in some studies their prevalence surpasses that of *C. albicans,* depending on the series and geographic location [33].

As previously mentioned, most cases of candiduria are asymptomatic, making fever and localized symptoms uncommon; pyuria is also infrequent. It is important to note that 46–80% of patients with candidemia present concomitant candiduria, and thus clinical monitoring is essential [34], as candiduria can be a symptom of a generalized infection. Nevertheless, unlike bacteriuria, the progression to candidemia remains low (1–8%), with higher rates in critically ill patients [2].

To aid in clinical decision-making, Fisher et al. [35] proposed a five-group classification: asymptomatic candiduria in healthy individuals; asymptomatic candiduria in outpatients with risk factors; asymptomatic candiduria in hospitalized patients with risk factors; symptomatic candiduria (cystitis, pyelonephritis, prostatitis, orchiepididymitis, fungal ball); and candiduria in unstable patients. They recommend initiating antifungal treatment in patients with evidence of renal infection, neutropenia, hemodynamic instability, or disseminated candidiasis. In all other cases, the clinical monitoring and management of predisposing factors (e.g., catheter removal or replacement, discontinuation of unnecessary antibiotics) are crucial. A follow-up urine culture is advisable to confirm spontaneous resolution, as persistent candiduria in at-risk patients may suggest occult infection. If candiduria persists despite these interventions, renal imaging should be performed to rule out local complications such as abscesses, anatomical abnormalities, or fungal balls requiring surgical intervention.

According to IDSA guidelines [16], candiduria is categorized as symptomatic or asymptomatic. In asymptomatic cases, the observation and mitigation of risk factors are recommended. In high-risk groups, such as neutropenic patients and low-birth-weight neonates, antifungal therapy is advised. The treatment of symptomatic candiduria depends on the clinical syndrome (cystitis/pyelonephritis, prostatitis/orchitis, or fungal ball). Fluconazole is the first-line therapy due to its high bioavailability (90%) and urinary concentration. Echinocandins are not recommended given their low renal excretion, although favorable outcomes have been reported in some cases [36]. For cystitis/pyelonephritis, the recommended regimen is fluconazole 200–400 mg/day for 14 days. Alternatives include flucytosine 25 mg/kg every 6 h for 7 days or bladder irrigation with amphotericin B deoxycholate (50 mg/L) via urinary catheter for 5 days. For prostatitis and orchiepididymitis, oral fluconazole 400 mg/day for 4 weeks is advised, with surgical drainage if necessary. In the case of fungal balls, treatment is similar, though surgical drainage is considered essential.

This study has certain limitations, including its observational, single-center design and the absence of a control group. Its single-center design may limit the generalizability of the results to other healthcare settings with different epidemiological, microbiological, or clinical practice characteristics. Nevertheless, the findings provide valuable information on the microbiological profile and associated factors in our center and may be comparable to institutions with similar characteristics. Furthermore, clinical context must be considered when differentiating between infection, which may require antifungal therapy and closer monitoring, and colonization. A major strength of this study lies in its large sample size, which allows for more robust data analysis.

## 5. Conclusions

This study highlights the importance of recognizing risk factors associated with candiduria to better identify at-risk clinical settings. Advanced age (>70 years), male sex, the presence of urinary catheters, and admission to ICUs, oncology, or surgical units emerged as significant predictors of candiduria, underscoring the need for clinical vigilance in these populations.

Although *C. albicans* remains the predominant species, a substantial proportion of non-albicans species, particularly *N. glabrata* and *C. tropicalis*, were also identified. This has direct implications for clinical management, as antifungal susceptibility varies by species. Therefore, individualized treatment strategies based on the specific *Candida* species isolated are essential for optimal patient outcomes.

## Figures and Tables

**Table 1:** Distribution of demographic and clinical variables in urine cultures, stratified by the presence or absence of *Candida* spp. (*n* = 111,656).

Variable	No *Candida* spp. (*n* = 108,619)	*Candida* spp. Present (*n* = 3037)	*p*-Value
Non-pediatric *	94,385 (86.90)	2986 (98.32)	<0.001
**Sex** (*Male*) *	49,647 (45.71)	1493 (49.16)	<0.001
**Urinary catheter** (*Yes*) *	27,527 (25.34)	1793 (59.04)	<0.001
**Requesting Service**			
*Primary Care*	1801 (1.66)	4 (0.13)	<0.001
*Outpatient Clinics*	2576 (2.37)	21 (0.69)	
*Medical Wards*	43,330 (39.89)	1521 (50.08)	
*Oncology*	1849 (1.70)	104 (3.42)	
*Surgical Wards*	4089 (3.76)	185 (6.09)	
*Intensive Care Unit (ICU)*	5887 (5.42)	361 (11.89)	
*Emergency Department*	49,087 (45.19)	841 (27.69)	

The table displays the distribution of pediatric status (non-pediatric), sex (male), and urinary catheter use (yes), distinguishing between urine cultures without (*n* = 108,619) and with (*n* = 3037) isolation of *Candida* spp. Additionally, the origin of the samples is broken down by requesting department (Primary Care, Outpatient Clinics, Medical Wards, Oncology, Surgical Wards, Intensive Care Unit [ICU], and Emergency Department), including frequency and percentage for each group, along with corresponding *p*-values for statistical significance. * *n* (%).

**Table 2:** Factors associated with the presence of *Candida* spp. in urine cultures.

	Bivariate Analysis			Multivariate Analysis		
**Variables [1]**	**ORc**	**95% CI (LL–UL)**	***p*-Value**	**ORa**	**95% CI (LL–UL)**	***p*-Value**
**Non-pediatric**	8.83	6.69–11.65	<0.001	11.83	8.96–15.63	<0.001
**Sex** *(Male)*	1.15	1.07–1.23	<0.001	–	–	-
**Urinary catheter** *(Yes)*	4.25	3.94–4.57	<0.001	–	–	-
**Sex x Urinary Catheter Interaction**						
*Male* vs. *Female (No catheter)*	–	–	-	1.00	0.89–1.12	0.990
*Male* vs. *Female (Yes catheter)*	–	–	-	0.82	0.74–0.90	<0.001
* Catheter Yes* vs. *No (Female)*	–	–	-	4.61	4.15–5.12	<0.001
*Catheter Yes* vs. *No (Male)*	–	–	-	3.78	3.38–4.22	<0.001
**Hospital Departments**						
*Primary Care (Reference category)*	1	-	-	1	-	-
*Outpatient Clinics*	3.67	1.26–10.71	<0.001	3.07	1.05–8.96	<0.05
*Medical Wards*	15.80	5.92–42.21	<0.001	9.49	3.55–25.38	<0.001
*Oncology*	25.33	9.31–68.89	<0.001	15.06	5.52–41.04	<0.001
*Surgical Wards*	20.37	7.55–54.94	<0.001	8.77	3.25–23.71	<0.001
*Intensive Care Unit (ICU)*	27.61	10.29–74.07	<0.001	10.05	3.73–27.04	<0.001
*Emergency Department*	7.71	2.89–20.62	<0.001	5.04	1.88–13.49	<0.001

**ORc**: Crude Odds Ratio. **ORa**: Adjusted Odds Ratio. **AUC** = 0.7457 (Area Under the Curve). This table presents the results of both bivariate and multivariate analyses of factors associated with the presence of *Candida* spp. Crude odds ratios (cORs) and adjusted odds ratios (aORs) are shown, along with their corresponding 95% confidence intervals (95% CI) and *p*-values. Variables analyzed include age group (non-pediatric vs. pediatric), sex, presence of urinary catheter, and the interaction between sex and catheter use. The impact of the hospital department is also evaluated, using Primary Care as the reference category.

## Data Availability

The original contributions presented in the study are included in the article; further inquiries can be directed to the corresponding authors.
